# Bigel Matrix Loaded with Probiotic Bacteria and Prebiotic Dietary Fibers from Berry Pomace Suitable for the Development of Probiotic Butter Spread Product

**DOI:** 10.3390/gels10050349

**Published:** 2024-05-20

**Authors:** Laura Tamašauskaitė, Vidmantė Minelgaitė, Aušra Šipailienė, Rimantė Vinauskienė, Viktorija Eisinaitė, Daiva Leskauskaitė

**Affiliations:** Department of Food Science and Technology, Kaunas University of Technology, Radvilenu pl 19, LT-50254 Kaunas, Lithuaniajukusike@gmail.com (V.M.);

**Keywords:** bigel, probiotic, prebiotic, co-encapsulation, food design

## Abstract

This study presents a novel approach to developing a probiotic butter spread product. We evaluated the prebiotic activity of soluble dietary fibers extracted from cranberry and sea buckthorn berry pomace with different probiotic strains (*Limosilactobacillus reuteri*, *Lacticaseibacillus paracasei*, and *Lactiplantibacillus plantarum*), uploaded selected compatible combination in the bigel matrix, and applied it in the probiotic butter spread formulation. Bigels and products were characterized by physical stability, rheological, textural properties, and viability of probiotics during storage at different conditions. The highest prebiotic activity score was observed in soluble cranberry (1.214 ± 0.029) and sea buckthorn (1.035 ± 0.009) fibers when cultivated with *L. reuteri*. The bigels loaded with probiotics and prebiotic fiber exhibited a significant increase in viscosity (higher consistency coefficient 40–45 Pa·s^n^) and better probiotic viability (>6 log CFU/g) during long-term storage at +4 °C temperature, surpassing the bigels loaded with probiotics alone. Bigels stored at a lower temperature (−18 °C) maintained high bacterial viability (above 8.5 log CFU/g). The butter spread enriched with the bigel matrix was softer (7.6–14.2 N), indicating improved spreadability. The butter spread product consistently met the required 6 log CFU/g for a functional probiotic food product until 60 days of storage at +4 °C temperature. The butter stored at −18 °C remained probiotic throughout the entire storage period, confirming the protective effect of the bigel matrix. The study’s results showed the potential of the bigel to co-encapsulate, protect, and deliver probiotics during prolonged storage under different conditions.

## 1. Introduction

Probiotic bacteria are major components of the human colon microbiota that have a symbiotic relationship with their host. According to the FAO/WHO, probiotic foods must contain more than 10^6^ CFU/g of probiotic bacteria to achieve sufficient health benefits for the host [1,2]. Even though probiotic microorganisms have been successfully incorporated into a variety of food products, they can face a wide range of harsh conditions through the food manufacturing process, transportation, and storage, as well as during passage through the gastrointestinal tract [3,4]. Therefore, it is essential to have sufficiently viable microorganisms after the consumption of probiotic food to achieve the expected beneficial effect on host health. In this respect, encapsulation of probiotics has been recognized as a successful way to preserve the cells under various environments [5,6]. Food products differ in their pH, ionic strength, water activity, and composition of nutrients. All these factors can increase or decrease the viability of probiotics in a product. In this context, the most significant attributes of the probiotic encapsulation system should be considered if these systems protect probiotic cells in food.

In recent years, two-phase gelation technology has shown significant progress in fundamental research and practical application for the encapsulation of bioactive materials [7]. This delivery system is made by combining two immiscible gels at a high shear rate, which afterward exists as a uniform dispersion hydrogel-in-oleogel called bigel [8]. As delivery systems of food bioactive substances, bigels have advantages over other carriers due to their high nutritional value, excellent functional properties, biocompatibility, biodegradability, and low toxicity. A crucial factor in the potential usage of bigels is the compatibility with lipid food matrices that could help expand probiotic food options. Butter is frequently used as a source of fat in the daily diet. As was previously reported, high fat content, as well as high density and texture, could create a more favorable environment for the prolonged survival of probiotics [9]. However, compared with other dairy-based food products, such as yogurt, cheese, fermented milk products, or powdered milk [10,11], butter is much less frequently supplemented with probiotics, highlighting the relevance of selecting bigel as a potential probiotics delivery system for butter matrix.

Regardless of the listed advantages, two-phase gelation technology is not yet widespread for encapsulating probiotics. Only a few studies focused on developing a bigel that can serve as a vehicle for the delivery of probiotics [12,13,14]. Probiotics inside bigels had much superior viability and were more tolerant of the gastric and intestinal environments than free cells. Nevertheless, long-term viability has not been achieved or described by storage or incorporating these systems into food products. Meanwhile, high probiotic survivability is essential for food products with a long shelf life to maintain the viability of probiotic strains above the recommended level (for therapeutic benefits) throughout the product’s storage [15]. Therefore, long-term bacterial viability is expected to be achieved by manipulating the composition of the bigel and/or by co-encapsulating probiotic bacteria with prebiotic compounds in the hydrogel phase. Co-encapsulation of probiotics and prebiotics, a relatively new concept, is used in such systems as calcium alginate carboxymethyl cellulose hydrogel beads, gelatin–gum arabic complex coacervates, and double emulsions [16,17,18] while never tested on the bigel matrix before. Prebiotic and probiotic combination, known as synbiotics, have been shown to improve probiotic proliferation since prebiotics serve as food for the beneficial bacteria and promote their growth, leading to a variety of health benefits [19].

The most extensively documented dietary prebiotics are galactants, fructans, *xylo*-oligosaccharides, isomalto-oligosaccharides, and lactulose [20]. Currently, interest has arisen in natural, renewable, and sustainable prebiotic sources, which can contribute to reducing the environmental footprint in addition to exhibiting prebiotic activity. Recent data indicate that vegetable, fruit, and berry by-products, generated mainly by the juice processing industry, have been recognized as a source of dietary fibers (soluble and non-soluble) with the following prebiotic capability [21]. It was found that sugar beet prebiotics increased the resistance of probiotics to bile salts, while chicory prebiotics performed a protective function in the intestinal environment [22]. Furthermore, there is evidence that the interaction of dietary fiber with other components of the food matrix, especially phenolic compounds, modulates the gut microbiota more effectively than the consumption of purified commercial fibers. It was previously reported that different berry pomaces are potential sources of phenolic substances, anthocyanins, sugars, dietary fiber, and other functional ingredients [23,24,25]. Water-soluble dietary fibers extracted from berry pomace rich in fructans, galactans, and phenolic compounds were investigated as potential prebiotics, selecting sea buckthorn and cranberry pomace, never previously studied in this context. 

Thus, the objective of this study was to find a suitable combination of water-soluble dietary fibers obtained from sea buckthorn and cranberry pomace and probiotic bacteria (*L. reuteri*, *L. plantarum*, and *L.s paracasei*) for co-encapsulation in a food-grade bigel system to increase probiotic viability with the following application in the butter spread product. 

## 2. Results and the Discussion

### 2.1. Prebiotic Activity (PA) of Soluble Fibers Extracted from Berry Pomace

Probiotic microorganisms have different preferences for different prebiotics, which plays a crucial role in promoting the proliferation of intestinal bacteria [26]. The prebiotic activity reflects the ability of a given substrate to support the growth and/or activity of probiotics capable of improving the health and well-being of the host [20]. The following section will analyze the prebiotic activity score of soluble dietary fibers extracted from various berry pomace or commercial prebiotic inulin (used as a control) paired with different probiotic strains.

**Figure 1:**
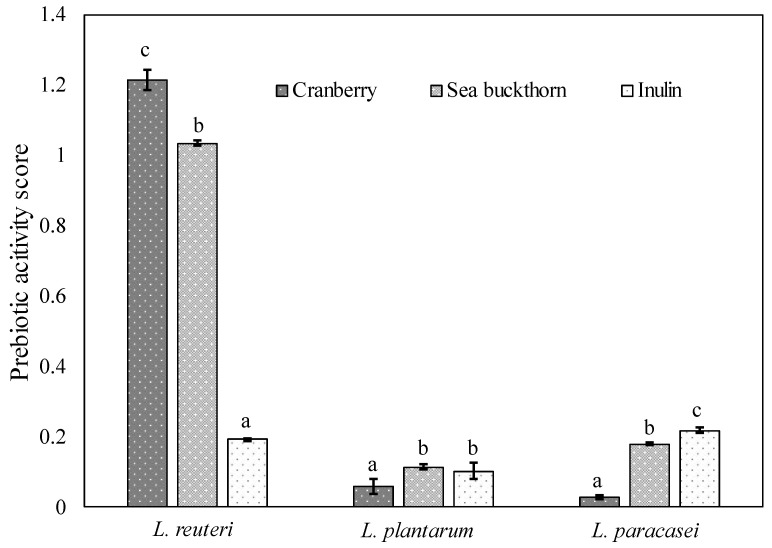
Prebiotic activity score of soluble dietary fibers extracted from berry pomace compared with inulin. The resulting values consist of average values with additional standard deviation. Statistical significance: *p* < 0.05.

The positive scores of prebiotic activity were found for all samples (Figure 1), indicating that tested soluble dietary fibers extracted from berry pomace can stimulate the growth of beneficial microorganisms and inhibit the growth of pathogenic bacteria, such as *Escherichia coli*. Authors in previous studies also found the prebiotic potential in Brazilian native fruits by-products such as cashew (for different *Lactobacillus* species) and jabuticaba (for *Lactobacillus* and *Bifidobacterium* species) [27,28], thus widening the variety of potential prebiotics.

We found that the prebiotic activity score varied quantitatively with the strains of probiotics tested. The highest prebiotic activity score was observed in the soluble cranberry (1.214 ± 0.029) and sea buckthorn (1.035 ± 0.009) fibers when cultivated with *L. reuteri*. This could be attributed to the mono- and di-saccharides presented in the composition of the fibers (concentrations indicated in the Materials and Methods section), as carbohydrates with lower molecular weight are considered more beneficial to the fermentation by intestinal bacteria [29]. It is also well known that lactic acid bacteria produce enzymes that are responsible for hydrolyzing glycosidic bonds in oligosaccharides and releasing α-glucose. It was also noticed that *L. reuteri* exhibits a high activity of α-galactosidase and β-galactosidase; however, enzymatic activity is strain-dependent [30].

Many studies have shown that structural properties of polysaccharides, such as molecular weight, composition of monosaccharides, and bond type, affect the prebiotic activity of the substrate [31]. *L. reuteri* exhibited selectivity for prebiotic dietary fibers, which followed the order cranberry > sea buckthorn > inulin, with a corresponding decrease in the di- and mono-saccharide content in these samples. It was previously reported that glucose is the primary carbon source needed for probiotic activity, thus prolonging their viability [32].

The prebiotic activity scores of various soluble dietary fibers extracted from berry pomace paired with *L. plantarum* and *L. paracasei* were considerably lower than those indicated for *L. reuteri*. Such a strain-dependent effect results from the phylogenetic diversity of each genus, causing different metabolic characteristics of microorganisms, which has been previously reported with different tested *Lactobacillus* strains [23,33].

It also should be mentioned that for *L. plantarum* and *L. paracasei* grown in a medium supplemented with soluble dietary fiber extracted from sea buckthorn pomace, the prebiotic activity scores were insignificantly higher or close to those determined for cells grown with commercial prebiotic inulin (used as control).

### 2.2. Bigels Loaded with L. reuteri and Soluble Dietary Fibers

Based on the results of the previously described prebiotic activity, the bigel containing *L. reuteri* and complementary soluble fiber extracted from cranberry and sea buckthorn pomace in the hydrogel phase was produced. Bigel with single-loaded *L. reuteri* was used as a control. Bigels properties were evaluated because these systems would be potentially used in chilled (0–4 °C) and frozen (−18 °C) food products. Thus, physical stability, rheological properties of bigel, and viability of loaded *L. reuteri* cells were examined during storage of loaded bigel at +4 °C and −18 °C.

#### 2.2.1. At +4 °C Temperature

**Figure 2:**
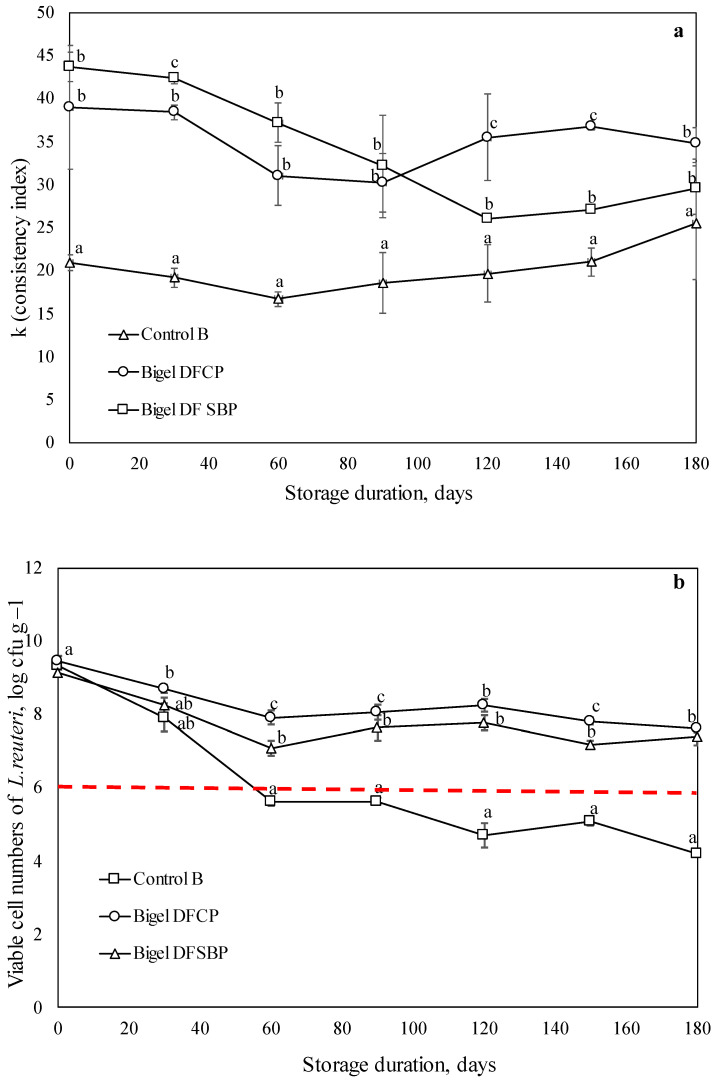
Changes in consistency index (**a**) and viability of *L. reuteri* cells (**b**) of bigels single loaded with probiotics and bigels loaded with probiotics and prebiotic dietary fibers during storage at +4 °C for 180 days; -------- minimum recommended viability level of probiotic [1,2]. The resulting values consist of average values with additional standard deviation. Statistical significance: *p* < 0.05. Control B—bigel loaded with *L. reuteri*; Bigel DFCP—bigel co-encapsulated with *L. reuteri* and soluble fibers extracted from cranberry pomace; Bigel DFSBP—bigel co-encapsulated with *L. reuteri* and soluble fibers extracted from sea buckthorn pomace.

The bigels had an off-white or slightly brownish color with a smooth, homogeneous texture and self-standing ability. The formation and stabilization of the bigels were based on the self-assembling of lipophilic (carnauba wax) and hydrophilic (collagen) gelling agents. No phase separation or discoloration was observed in all bigel formulations throughout the storage period (180 days), indicating appreciable stability. The apparent viscosity of the bigels prepared from all formulations showed a significant decrease with increasing shear rate, suggesting a shear-thinning behavior that agrees with previous studies [34]. The consistency indices of bigel loaded with probiotics were lower than those of bigels loaded with probiotics and prebiotic dietary fibers with slightly varying values during the whole period of storage (Figure 2a). Most probably, soluble fiber addition affected the high level of entanglement of molecules and raised the viscosity. Better rheological characteristics in emulsion gel samples with soluble dietary fibers have been previously attributed to cross-linking between soluble dietary fiber and proteins [34], suggesting occurring collagen–dietary fiber interactions in our system.

One factor determining the suitability of the delivery system is stability during storage. The absence of major textural changes during bigel storage confirms the sufficiently long shelf life of bigels. However, it is essential to verify whether the structure of the bigel and prebiotic inclusion have a protective effect on probiotics immediately after preparation and during long-term storage.

In the initial time point, the viable cell numbers of all tested samples varied between 9.15 and 9.46 log CFU/g with no significant difference among the sample groups. This indicates that *L. reuteri* retained resilience throughout the entire bigel preparation process, considering that homogenization is often described as a factor that may reduce the viability of probiotics due to their sensitivity to the shear stress applied [35]. The single-loaded *L. reuteri* bigel sample retained the minimum recommended viability for probiotics for one-third (60 days) of the total storage period (180 days). This demonstrates that the double-gel structure of bigel worked as a physical barrier to protect probiotics from the environment. First, the probiotics were immobilized in the hydrogel phase through the self-assembly of collagen fibrils by the multiple non-covalent intermolecular interactions [36]. Additionally, the oil phase in the bigel composition was transformed into the platelet-like crystalline network by the presence of carnauba wax [37]. It is assumed that this double-gel network helped to prevent probiotic migration from phase to phase, as it has already been reported for semi-solid and liquid emulsions [10]. However, our previous statement that encapsulation does not ensure microorganisms’ total viability during prolonged storage has been confirmed. Viable cell numbers in single probiotic-loaded bigel were detected at the level 4.21 log CFU/g, showing >5 log cycle reduction by 180 days of storage. The most probable reason for this phenomenon is a deficiency of nutrients in the environment necessary for the growth of probiotics.

Throughout the storage period of up to 180 days, *L. reuteri* viable cell numbers were found to be 7.41 and 7.61 log CFU/g (Figure 2b) when loaded into bigels with prebiotic dietary fibers. Such a prebiotic-rich micro-environment around probiotics plays an important role in maintaining probiotic viability as co-encapsulated *L. reuteri* did not reduce below the minimum recommended level over a long-term period of storage. These results were similar to those found by other authors stating that the addition of prebiotics such as inulin, fructooligosaccharide, sugar beet, oat, chicory, and hi-maize starch significantly improved the survival rate of different probiotic bacteria in various carrier matrices [18,22,38]. The obtained results demonstrate that co-encapsulation of *L. reuteri* and soluble dietary fibers extracted from cranberry and sea buckthorn pomace is beneficial for the storage stability of probiotics.

#### 2.2.2. At −18 °C Temperature

**Figure 3:**
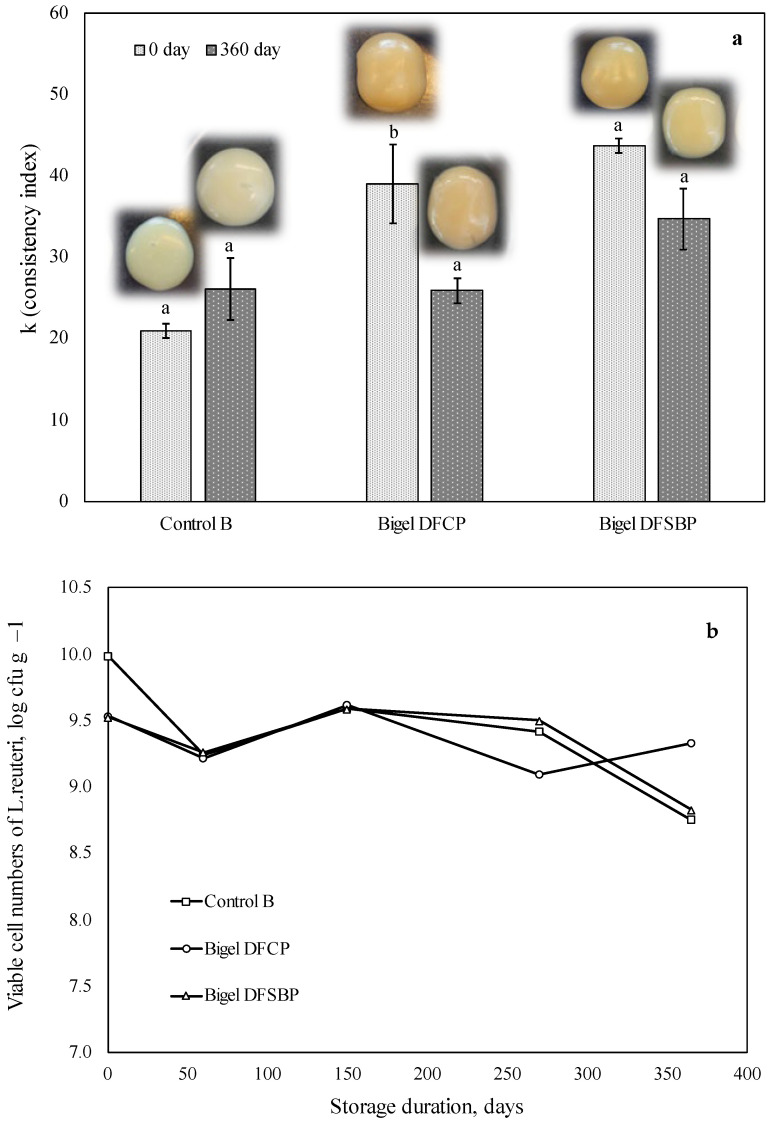
Changes in consistency index (**a**) and viability of *L. reuteri* cells (**b**) of bigels single loaded with probiotics and bigels loaded with probiotics and prebiotic dietary fibers during storage at −18 °C for 360 days. Images represent the visual appearance of the bigel systems immediately after preparation and after 360 days of storage. The resulting values consist of average values with additional standard deviation. Statistical significance: *p* < 0.05. Control B—bigel loaded with *L. reuteri*; Bigel DFCP—bigel co-encapsulated with *L. reuteri* and soluble fibers extracted from cranberry pomace; Bigel DFSBP—bigel co-encapsulated with *L. reuteri* and soluble fibers extracted from sea buckthorn pomace.

The rheological properties of bigels are a vital factor reflecting their freeze–thaw stability. It has already been described above that at the beginning of storage, single-loaded *L. reuteri* bigel had the lowest consistency index compared to the systems containing prebiotics. A slight decrease in consistency coefficients was observed in the systems with prebiotics during further storage, which remained unaffected until the end of the experiment (Figure 3a). Notably, there were no visible signs of phase separation or other signs of destabilization (Figure 3a), indicating resistance of the formed dual-gel structure to the active big ice crystal growth during freezing. Our results agree with other reports demonstrating that stearic acid-based oleogel with konjac glucomannan gelatin-based binary hydrogel exhibited suitable stability with high freeze–thaw resistance [39].

Probiotic storage at negative temperatures could also positively affect their viability [40], which needs to be verified during this study. Therefore, while keeping the bigels at −18 °C, we have extended their storage duration for 360 days. At a starting concentration of approximately 9.5–10.0 log cfu/g, *L. reuteri viable* cells remained relatively stable at −18 °C, registering < 1–1.5 log cycle reduction by the end of the storage. It is essential to note that probiotic viability remained above 8.5 log cfu/g throughout the whole storage period (Figure 3a), which is in accordance with recommended levels of viable probiotic cell counts in food at the time of consumption [1,2]. First, the results demonstrate that double-gel protects probiotic bacteria from membrane damage and osmotic imbalance caused by ice crystals during storage and thawing processes [18].

It was also found that sub-zero temperature positively affected the *L. reuteri* viability, as it was significantly higher compared to bigels stored at +4 °C temperature. Such temperature-related probiotic viability was previously reported for probiotic microcapsules made with gelatin–gum arabic complex coacervate (as coating material) [17]. In accordance with our findings, the authors identified that lower temperature exhibits more positive effects on encapsulated probiotic viability. Higher temperatures could intensify metabolic and cellular activities, causing nutrient depletion and eventual cell death [41].

In contrast to the bigels stored at higher temperatures (+4 °C), the effect of prebiotics was not as significant as their effect at lower temperatures. This phenomenon could be explained by the fact that at lower temperatures, probiotics are in a state of suspended animation, preventing proliferation, thus making added prebiotics less functional.

### 2.3. Butter Spread Product with Loaded Bigel

To produce a probiotic butter spread product using bigels as a carrier of microorganisms, it is crucial to evaluate the effect of the bigel addition (10% *w*/*w*) on the properties of the product. In butter, even minor compositional changes can affect the organization of the fat crystal network, causing changes in the properties of the product [42]. It is essential to evaluate these changes when modifying the traditional composition, as they may affect consumer acceptance (mouthfeel effect) and the overall quality of the final product. The following section will identify the textural and rheological properties of the butter spread product made with the addition of loaded bigel stored at different temperatures (4 °C and −18 °C). The obtained information will provide further insight into the effect of the compositional reformulation on the functionality of the butter.

#### 2.3.1. Textural and Rheological Properties

Textural and rheological properties of high-fat products are qualitative parameters that depend on solid/liquid fat fraction, fat crystal network, and moisture content [43]. We found that butter spread product samples showed significant differences in firmness, as bigel-loaded butter spread was found to be softer (Table 1), which suggests that this sample had higher spreadability (preferred by consumers, more convenient to use). Such an inverse relationship between spreadability and hardness was previously reported by other authors [43]. It can be assumed that lower firmness values were obtained due to the difference in fatty acids composition caused by bigel addition to the high-fat butter. Moreover, in addition to the fat-compatible oleogel phase, bigel contains a hydrogel phase, which is also distributed in the structure of butter during its manufacturing. Such reformulation may have impacted randomly arranged fat crystal networks, causing weaker texture and more soft butter spread product [44].

Over a 6-month storage period, the data collected show a significant decrease (*p* < 0.05) in the firmness values, which was higher in butter containing bigel when compared to the control (Table 1). Such a time-dependent softening process could be related to the polymorphic stability of the butter, driven by the transition of fat crystal shapes (α, β′, and β) from one form to another [45]. Different authors raised the hypothesis that butter samples with olein addition became softer and more easily spreadable because of the formation of the most unstable polymorphic α form [46].

Concerning the rheological properties, solid-like behavior (G′ > G″) was observed in both samples under oscillatory deformations. Control and bigel-loaded butter spread showed tanδ < 1 (0.20–0.48) with constant storage (G′) and loss (G″) modulus across the whole frequency range, suggesting a well-structured network. As the storage time was increased, the results showed imperceptible changes of storage modulus G′ for both butter spread products regardless of the storage temperature. It also found that the G′ value directly correlated with the sample’s firmness, in correspondence with a study performed with *Lactobacillus helveticus* butter [44].

#### 2.3.2. Viability of Probiotic

A positive effect on the textural properties of butter after the addition of a bigel additive with no adverse effect on stability was found. At the same time, the next step was to evaluate whether bigel could be used as a carrier of *L. reuteri* in the production of probiotic fat-based products. Co-encapsulation of *L. reuteri* in combination with soluble dietary fiber extracted from berry pomace was found to increase the viability of the probiotic, with no apparent significant differences between fiber types. This is why prebiotic dietary fibers extracted from sea buckthorn pomace were chosen for this experiment stage, co-encapsulated with *L. reuteri* in the hydrogel phase of the bigel, and used as such in the butter spread product formulation. The probiotic butter spread product was stored at different temperatures, and *L. reuteri* viability was determined monthly.

**Figure 4:**
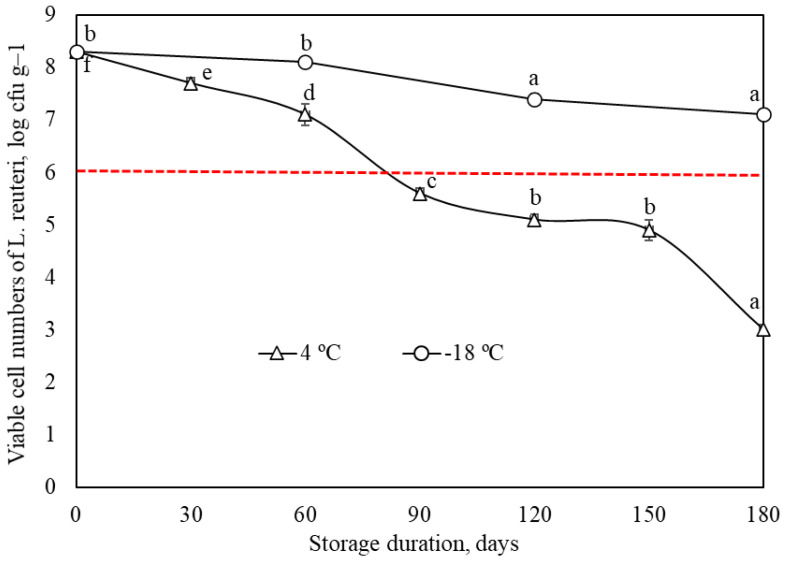
Viability of *L. reuteri* cells of butter spread product loaded with co-encapsulated bigel during storage at 4 °C and −18 °C for 180 days. The resulting values consist of average values with additional standard deviation. Statistical significance: *p* < 0.05.

The number of probiotic bacteria in the product is a crucial index for qualifying the product as a probiotic. Compared with bigels, the butter spread product initially showed lower probiotic concentration from the day of manufacture (1–1.5 log cycle), indicating that mechanical mixing during the incorporation of bigels in the butter matrix negatively affected bacteria viability. Subsequent storage of the butter at +4 °C temperature showed a gradual decrease in bacterial content comparable to that observed during storage of the bigels. In this case, the lower initial viable *L. reuteri* cells resulted in the butter spread product meeting the required 6 log CFU/g for a functional probiotic food product only until 60 days of storage (Figure 4). The obtained duration was longer than those reported for probiotic butter produced with encapsulated *Bifidobacterium bifidum* [47] and for probiotic butter with *Lactobacillus acidophilus* and *Bifidobacterium bifidum* [48]; however, this does not fully meet our expectation. At the same time, other authors managed to keep the probiotics viable for a more extended period by using different encapsulation techniques in such product as bigel butter spread [49].

As expected, further storage of the butter spread product resulted in the reduction in probiotics to a level where the product cannot be classified as a probiotic product (<6 log CFU/g). The findings align with a previously reported decreasing manner of co-encapsulated *L. plantarum* during storage of white cheese samples (at 4 °C, 61 days) [50]. The authors identified the main reasons for this decline as high salt concentration and relatively low pH of the cheese. Regarding a significantly more significant decrease in the encapsulated probiotic content after incorporation into the butter matrix, the competitive effect of starter culture or metabolic products produced by lactic acid bacteria may have contributed to the inhibition of *L. reuteri* growth. This assumption is reasonable in the case of progressive migration of probiotics from the bigel matrix to the butter medium.

Different trends in bacterial viability were observed when the butter spread product was stored at −18 °C. During the entire product storage period of 180 days, the number of probiotic bacteria decreased slightly to 7.1 log CFU/g. So, according to existing requirements, the product remained probiotic during the entire storage period. We therefore assume that bigel maintained the integrated structure even under destructive factors, such as thermal stress, osmotic pressure, oxidative stress, and mechanical forces caused by ice crystal formation [51]. This allows the protection of cells and prolongs their shelf life from undesirable environmental factors. Additionally, the obtained results reveal the relevance of storage temperature rather than prebiotic incorporation. Processes that negatively affect microorganisms’ viability are slowed down at lower temperatures, allowing the probiotics to remain viable [51].

## 3. Conclusions

Never previously investigated in this context, soluble dietary fibers extracted from cranberry and sea buckthorn berry pomace were evaluated as potential prebiotics with different probiotic strains (*L. reuteri*, *L. paracasei*, and *L. plantarum*). The positive scores of prebiotic activity found for all samples indicate that tested soluble dietary fibers extracted from berry pomace can stimulate the growth of beneficial microorganisms and inhibit the growth of pathogenic bacteria, such as *E. coli*. However, an exceptionally high prebiotic index was found in soluble cranberry (1.214 ± 0.029) and sea buckthorn (1.035 ± 0.009) fibers in combination with *L. reuteri.*

Co-encapsulation of *L. reuteri* and soluble dietary fibers affected the increase in viscosity (higher consistency indices 40–45 Pa·s^n^) and better viability of encapsulated probiotics (>6 logCFU/g) throughout storage at +4 °C temperature compared to single-loaded *L. reuteri*. In contrast, in bigels stored at a lower temperature (−18 °C), the positive effect of prebiotics on bacteria viability was not as significant as the positive effect of lower temperature.

Bigel-loaded butter spread showed a significantly lower firmness value, which suggests that this sample had higher spreadability. Over a 6-month storage period, time-dependent butter softening was obtained. However, this did not negatively affect either the stability of the butter or the overall texture. The probiotic butter spread product initially showed lower probiotic concentration from the day of manufacture (1–1.5 log cycle), indicating that mechanical mixing during the incorporation of bigels in the butter matrix had a negative effect on bacteria viability. This resulted in the butter spread product meeting the required 6 log CFU/g for a functional probiotic food product until 60 days of storage. In contrast, probiotic viability was higher than 7 log CFU/g throughout a 180-day period of storage at −18 °C temperature, showing that bigel maintained the integrated structure even under destructive factors caused by ice crystal formation.

Overall, the results showed that bigel matrix and co-encapsulation combined with prebiotics could protect probiotics and increase their viability. Such a delivery system could be successfully applied to high-fat food products.

## 4. Materials and Methods

### 4.1. Materials

Collagen (bovine, I and III type mixture; 90% of protein) was obtained from MyProtein (Manchester, UK). Sunflower oil and olive pomace oil (containing saturated (10.8 g/100 mL), mono-unsaturated (32.8 g/100 mL), and polyunsaturated (48.3 g/100 mL) fatty acids) were purchased from the local supermarket (Basso, San Michele di Serino, Italy) and used as such. Carnauba wax (melting point 82–84 °C) obtained from Sigma-Aldrich (St. Louis, MO, USA) was used as an oil structuring agent. Pasteurized cream of 35% (Dvaro, Pieno žvaigždės, Lihuania) fat was used for butter manufacturing.

### 4.2. Methods

#### 4.2.1. Extraction of Soluble Prebiotic Dietary Fiber 

The berry pomaces were obtained using frozen berries that were donated by the Fudo Company (Kaunas, Lithuania). The berries were thawed and pressed in a Philips HR1880/01 juicer. Obtained cranberry (*Vaccinium macrocarpon* Ait) and sea buckthorn berry (*Hippophae rhamnoides* L.) pomace was dried to a moisture content of 7–9% by using the hot air (35–40 °C, 48–72 h) drying method. The dried pomace was cooled, weighed, and stored in sealed packages in a well-ventilated room with a relative humidity of no higher than 75% and an ambient temperature not exceeding 20 °C for up to 4 months. Before usage, dry pomace was milled to 0.2–0.25 mm particles. Pomace powders were mixed with water in a ratio of 1:10, stirred for 10–15 min, and centrifuged at 8000–10,000 rpm for 15 min. The separated water-soluble fraction was mixed with ethanol in a ratio of 5:95 and stirred for 5–10 min. After filtering, the sediments were separated and dried by using freeze-drying (−50 °C, 0.5 mbar, 24–48 h). The saccharide profile in soluble dietary fibers was as follows: cranberry (oligosaccharides—11.1 g/100 g dw; mono- and di-saccharides—20.5 g/100 g dw, of which sucrose—0.5 g/100 g dw, glucose—5.4 g/100 g dw, and fructose—2.8 g/100 g dw), sea buckthorn (oligosaccharides—6.1 g/100 g dw; mono- and di-saccharides—9.1 g/100 g dw, of which sucrose—0.85 g/100 g dw, glucose—1.5 g/100 g dw, and fructose—1.1 g/100 g dw).

#### 4.2.2. Prebiotic Activity (PA)

The prebiotic activity of various soluble dietary fibers extracted from berry pomace or commercial prebiotic inulin (known as prebiotic and used as a control) paired with different probiotics was examined. Commercial strain *L. paracasei subsp. paracasei ATCC^®^ BAA*-52 was used in the experiment, while *L. reuteri* 182 and *L. plantarum F1* were provided by the KTU Food Institute collection (Kaunas, Lithuania). The strains were isolated from spontaneous sourdough bread and identified at the KTU Food Institute. 

The assay was performed according to Huebner et al. (2007) by adding 1% (*v*/*v*) of an overnight culture of each probiotic strain to separate tubes containing MRS broth with 1% (*w*/*v*) glucose or 1% (*w*/*v*) soluble dietary fibers extracted from berry pomace or inulin. The cultures were incubated at 37 °C in an ambient atmosphere. After 0 and 24 h of incubation, samples were enumerated on De Man, Rogosa (MRS), and Sharpe agar (Liofilchelm, Roseto degli Abruzzi, Italy). In addition, overnight, *E. coli* ATCC 25,922 bacteria were added at 1% (*v*/*v*) to separate tubes containing M9 broth with 1% (*w*/*v*) glucose or 1% (*w*/*v*) prebiotic. The cultures were incubated at 37 °C in an ambient atmosphere and enumerated on Plate Count Agar (PCA, Liofilchelm, Roseto degli Abruzzi, Italy) after 0 and 24 h of incubation. Each assay was replicated three times. The prebiotic activity score was determined using the following equation:

$$\begin{array}{l} PA\\ =\left[\frac{\left(Probiotic\;lg\;CFU/ml\;on\;the\;prebiotic\;at\;24\;h-Probiotic\;lg\;CFU/ml\;on\;the\;prebiotic\;at\;0\;h\right)}{(Probiotic\;lg\;CFU/ml\;on\;the\;glucose\;at\;24\;h-Probiotic\;lg\;CFU/ml\;on\;the\;glucose\;at\;0\;h)\;}\right]\\ -\left[\frac{\left(E.\;coli\;lg\;CFU/ml\;on\;the\;prebiotic\;at\;24\;h-E.\;coli\;lg\;CFU/ml\;on\;the\;prebiotic\;at\;0\;h\right)}{(E.\;coli\;lg\;CFU/ml\;on\;the\;glucose\;at\;24\;h-E.\;coli\;lg\;CFU/ml\;on\;the\;glucose\;at\;0\;h)\;}\right] \end{array}$$

#### 4.2.3. Probiotic Preparation

The probiotic (*L. reuteri*) strains were separately activated using MRS broth and incubated at 37 °C for 22 h aerobically. After incubation, the probiotic cells were obtained by centrifugation at 6000 rpm for 10 min at +4 °C and washed with sterile saline water. The obtained probiotic cell suspension contained no less than 1.3 × 10^11^ CFU/mL of viable cells and was used in the bigel preparation.

#### 4.2.4. Bigel Preparation

Bigel loaded with probiotics (as control) and bigels loaded with probiotics and prebiotic dietary fibers were prepared. For this purpose, the water phase was prepared by dissolving 60 g/100 g of collagen and 1.34 g/100 g of soluble dietary fiber in distilled water and incubating at 85 °C for 30 min, continuously mixing. The water phase was prepared under the same conditions for the control bigel, the only difference being that no soluble fiber was added. For the oleogel phase, 9 g/100 g of carnauba wax (as a gelator) was dissolved in vegetable oil and incubated at 85 °C for 30 min. The concentration of collagen and carnauba wax was previously selected in preliminary tests as being suitable for stabilizing the bigel and not adversely affecting the sensory characteristics of the bigel.

The resulting oil and water phases were homogenized in two stages. Firstly, oil and water phases were mixed at a ratio of 25:75 and homogenized for 60 s at 15,000 rpm, maintaining the temperature at 85 °C. The mixture was cooled to 55 °C, 1 mL/100 g of pre-prepared probiotic suspension was added, and the mixture was additionally homogenized at 11,000 rpm for 60 s. Immediately after homogenization, the mixture was transferred to the ice bath to induce gelation of both phases and was stored at +4 °C (for 180 days) or −18 °C (for 360 days).

#### 4.2.5. Probiotic Butter Spread Preparation

Freshly prepared bigel uploaded with *L. reuteri* and dietary fiber extracted from sea buckthorn pomace was further used to prepare butter spread. The butter was manufactured at the Kaunas University of Technology Dairy Plant (Kaunas, Lithuania). The cream was churned (Milky, Buttermaschine FJ32, Althofen, Austria) until it formed butter grains and then thoroughly washed with water to remove the whey. After removing most free water, the butter was gently mixed (speed—800 rpm at 25 °C temperature) with the initially prepared bigel matrix (10% *w*/*w*) uploaded with probiotics and dietary fibers. It was called probiotic butter spread. Butter mixed with the same amount of bigel matrix without probiotic cells was used as a control. The probiotic butter spread and control samples were packed in 100 g plastic containers and kept refrigerated at 5 °C and −18 °C during the analysis period.

#### 4.2.6. Bigel Characterization

##### Physical Stability

Formulated bigels were kept at 4 °C in tightly sealed bags for 180 days, and their visual appearance was recorded each month to assess the storage stability of the samples. The visual appearance of the phase separation was considered the beginning of the destabilization of the bigels.

##### Rheological Properties

Rheological properties of the bigel samples were evaluated by shear sweep and frequency sweep tests at 25 °C using a rheometer (MCR 92, Anthon Paar, Sttutgard, Germany) with a plate–plate system PP25 (diameter 20 mm, gap 1 mm). The flow behavior was estimated over a shear ranging from 0.01 to 500 s^−1^. Data were analyzed using the Herschel–Bulkley model, and the viscosity coefficient (ĸ) and flow index (n) were calculated. The limit of the linear viscoelastic (LVE) area was confirmed by the amplitude sweep test before the frequency sweep test, and a shear strain value of 0.1% was determined for the LVE region. In the frequency sweep test, the storage (G′) and loss moduli (G′) were measured, and the angular frequency was changed from 0.1 to 100 rad/s at 25 °C. Before the tests, the bigels, which were stored at +4 °C, were removed from the fridge and left at room temperature for 1 h before testing. In contrast, bigels that were kept at −18 °C temperature were placed in the fridge (for +4 °C temperature) for 12 h (thawing process) and later removed from the refrigerator for 1 h before testing at room temperature.

##### Viability of Probiotic Cells

The viability of probiotic cells in bigel samples was measured every month of the storage. The viable counts of *L. reuteri* were determined at all sampling points following EN ISO 15214:1998 Standard [52]. A total of 1 g of the bigel was weighed into a tube containing pre-warmed (37 °C) 9 mL of sterile saline water, and serial dilutions were made so that the number of colonies per plate was between 15 and 300. The viable counts of *L. reuteri* were evaluated by the pour plating method of 1 mL of preparation in MRS Agar with Tween 80. All samples were plated in quadruplicate. The plated *Petri* plates were incubated at 37 °C temperature for 72 h. Viable cell numbers were calculated as log_10_ CFU/1 g.

#### 4.2.7. Characteristics of the Probiotic Butter Spread Product

##### Rheological Characteristics

Frequency sweep tests at 25 °C using a rheometer with a plate-to-plate system (gap 2 mm) were carried out as described above.

##### Textural Analysis

The analysis used a 20 mm metallic cylindrical probe in a TA.XT plus Texture Analyzer (Stable Micro Systems, London, UK) equipped with a 5 kg load cell. The test performed was a compression test, in which the probe penetrated a distance of 10 mm from the sample’s surface with a test speed of 1 mm/s. Hardness (the maximum force of penetration expressed as Newtons) was obtained from the force–deformation curve.

##### The Viability of Probiotic Cells

Probiotic cells’ viability during storage of butter spread samples was measured as described above.

#### 4.2.8. Statistical Analysis

All analyses were carried out in triplicate. The results are presented as the mean ± standard deviation. A *p*-value of <0.05 was used to indicate significant differences between the mean values determined by an analysis of variance (ANOVA) using Statistica 12.0 (StatSoft, Inc., Tulsa, OK, USA, 2013). For sensory evaluation, scores were submitted to the ANOVA with product, sex, and dysphagia (yes/no) as fixed factors and participants as random factors. Interactions were removed from the model as they were found to be not significant.

## Figures and Tables

**Table 1:** Changes in probiotic and non-probiotic butter spread product during storage at +4 °C and −18 °C.

Storage Duration, Days	Butter (Control)		Butter Spread Product with Loaded Bigel	
	Storage Modulus G′, Pa·10^5^, at 1 Hz,	Firmness, N	Storage Modulus G′, Pa·10^5^, at 1 Hz,	Firmness, N
	At 4 °C Temperature			
0	1.64 ± 0.04 ^bB^	18.8 ± 1.4 ^bD^	1.49 ± 0.08 ^aAB^	14.2 ± 3.2 ^aD^
30	1.28 ± 0.12 ^aA^	20.2 ± 1.4 ^bE^	1.55 ± 0.30 ^bAB^	14.2 ± 3.2 ^aCD^
60	1.81 ± 0.03 ^bBCD^	20.2 ± 1.5 ^bE^	1.48 ± 0.07 ^aAB^	13.8 ± 1.7 ^aCD^
90	1.78 ± 0.05 ^bBCD^	14.6 ± 1.6 ^bA^	1.55 ± 0.07 ^aAB^	10.3 ± 1.1 ^aB^
120	1.85 ± 0.17 ^bCD^	16.6 ± 1.6 ^bC^	1.57 ± 0.04 ^aB^	8.9 ± 1.3 ^aAB^
150	1.85 ± 0.03 ^bD^	15.6 ± 1.7 ^bB^	1.52 ± 0.01 ^aAB^	8.1 ± 0.9 ^aAB^
180	1.81 ± 0.08 ^bBCD^	14.2 ± 2.2 ^bA^	1.49 ± 0.02 ^aAB^	7.6 ± 0.6 ^aA^
	At −18 °C Temperature			
0	1.64 ± 0.04 ^bBC^	18.8 ± 1.4 ^bD^	1.49 ± 0.08 ^aAB^	14.2 ± 2.8 ^aE^
30	1.39 ± 0.07 ^aA^	16.2 ± 0.9 ^bC^	1.55 ± 0.17 ^bB^	10.0 ± 0.8 ^aD^
60	1.75 ± 0.08 ^bC^	18.4 ± 2.1 ^bD^	1.48 ± 0.09 ^aAB^	14.7 ± 2.5 ^aE^
90	1.70 ± 0.16 ^bBC^	9.7 ± 1.5 ^bA^	1.35 ± 0.02 ^aAB^	7.8 ± 0.4 ^aCD^
120	1.70 ± 0.00 ^bBC^	11.5 ± 1.0 ^bB^	1.36 ± 0.09 ^aAB^	6.9 ± 0.3 ^aBC^
150	1.74 ± 0.09 ^bBC^	10.5 ± 0.8 ^bAB^	1.32 ± 0.10 ^aA^	5.3 ± 0.8 ^aAB^
180	1.72 ± 0.13 ^bBC^	9.2 ± 0.6 ^bA^	1.32 ± 0.12 ^aA^	4.2 ± 0.9 ^aA^
Visual image of the butter spread samples	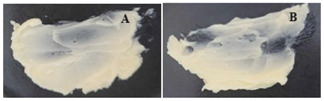		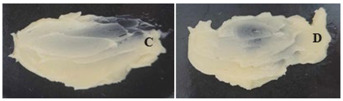	

Values are expressed as mean ± standard deviation. Different superscript small letters in the same row indicate significant (*p* < 0.05) differences between samples; different superscript capital letters in the same column indicate significant (*p* < 0.05) differences during storage. The images show the appearance of the butter after storage for 180 days at the appropriate temperature: A—control butter spread products stored at +4 °C temperature; B—probiotic butter spread products stored at +4 °C temperature; C—control butter spread products stored at −18 °C temperature; D—probiotic butter spread product stored at −18 °C temperature.

## Data Availability

The data presented in this study are openly available in the article.
